# Phonetic categorization relies on motor simulation, but combinatorial phonological computations are abstract

**DOI:** 10.1038/s41598-023-28099-w

**Published:** 2023-01-17

**Authors:** Iris Berent, Peter J. Fried, Rachel M. Theodore, Daniel Manning, Alvaro Pascual-Leone

**Affiliations:** 1 Department of Psychology, Northeastern University, Boston, MA USA; 2 Berenson-Allen Center for Noninvasive Brain Stimulation, Beth Israel Deaconess Medical Center, Boston, MA USA; 3 Department of Neurology, Harvard Medical School, Boston, MA USA; 4 Department of Speech, Language, and Hearing Sciences, University of Connecticut, Storrs, USA; 5 Hinda and Arthur Marcus Institute for Aging Research, Deanna and Sidney Center for Memory Health, Hebrew SeniorLife, Boston, MA USA; 6 Guttmann Brain Health Institute, Barcelona, Spain

**Keywords:** Human behaviour, Cognitive neuroscience, Language

## Abstract

To identify a spoken word (e.g., *dog*), people must categorize the speech steam onto distinct units (e.g., contrast *dog/fog*,) and extract their combinatorial structure (e.g., distinguish *dog/god*). However, the mechanisms that support these two core functions are not fully understood. Here, we explore this question using transcranial magnetic stimulation (TMS). We show that speech categorization engages the motor system, as stimulating the lip motor area has opposite effects on labial (*ba/pa*)- and coronal (*da/ta*) sounds. In contrast, the combinatorial computation of syllable structure engages Broca’s area, as its stimulation disrupts sensitivity to syllable structure (compared to motor stimulation). We conclude that the two ingredients of language—categorization and combination—are distinct functions in human brains.

## Introduction

Like many other species, humans rely on a vocal system of communication^1^. But the human capacity for language may go beyond the vocal command of speech. Language is a discrete, combinatorial system^2,3^. The combination of speech sounds conveys linguistic information, much like the sequencing of nucleotide bases carries genetic information. It is this combinatorial function that allows speakers to form and comprehend novel linguistic forms that they have never heard before^2,3^.

**Figure 1:**
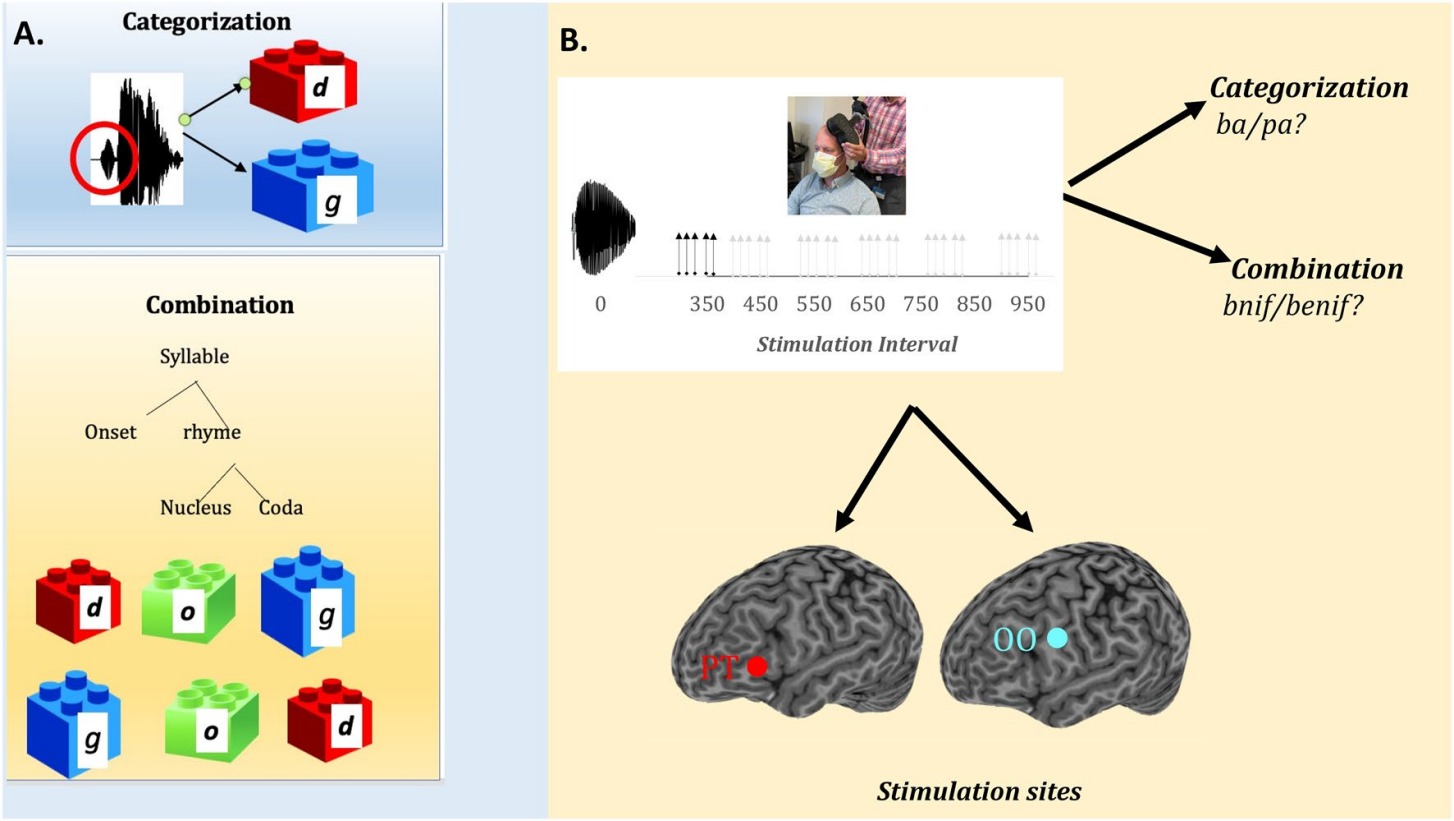
The two core ingredients of language. (**A**) Illustrates the two computations of interest—speech categorization (e.g., as *d* or *g*) and combination of speech sounds into syllables (e.g., as *dog* or *god*). (**B**) Illustrates how these two functions were gauged in our TMS studies. In each experiment, participants were administered a brief 50 Hz TMS train (5 pulses) at seven distinct intervals occurring after the onset of the auditory stimulus. Stimulation, in turn, targeted either the primary motor representation of the orbicularis oris muscle (OO) or the pars triangularis of the left frontal operculum (PT). In Experiment 1, participants categorized the ambiguous auditory stimulus; in Experiments 2, they identified the number of syllables (as a test of the combinatorial function). Note: written informed consent was obtained to use and publish the person’s image (in Fig. 1b).

Categorization and combination, then, are core ingredients of language (see Fig. 1A). To understand spoken language, people must categorize the speech stream into distinct functional units (e.g., as either *b* or *p*), and extract their combinatorial structure (e.g., *bat* or *tab*). However, the brain mechanisms that support these two key computations are not fully understood.

One hypothesis is that language comprehension engages the cognitive and brain mechanisms that mediate its production^4^, in line with the “embodied cognition” view^5^. Thus, to identify a speech unit (e.g., *b*, in *bag*), listeners must simulate its articulation, as if they were about to utter that sound aloud^4^. Indeed, when people hear a linguistic sound (e.g., the labial sound *b*), they spontaneously activate the brain motor area that controls its articulation (e.g., the lip motor area)^6^, and when activity in that brain motor area is altered (by electro-magnetic or mechanical stimulation), so is also the perception of that specific sound^7–17^.

Just like the case for sound categorization, the formation of combinatorial structure for spoken language (i.e., phonology^18,19^) could be likewise governed by the motor system. If so, to assemble syllable structure (e.g., *blog* vs. *lbog*), speakers would tacitly articulate the sound sequence. Difficulty of articulation would then determine the frequency of the syllables across languages^20^. Indeed, phonological combinations that are preferred across languages are typically ones that are also easier for the articulatory system to produce (e.g., *blog* > *lbog*)^21^. But whether the motor system does, in fact, mediate the formation of combinatorial structure is uncertain.

The correlation between articulatory and linguistic preferences does not settle this question, as it does not prove that the motor system has a *causal* role in the combinatorial function of speech sounds. In fact, existing evidence suggests that, unlike the categorization of speech sounds, perceiving their combination (i.e., phonology) is mostly impervious to disruption of the articulatory motor system^22,23^, and instead recruits the pars triangularis of the frontal operculum (part of Broca’s area)^24^. Moreover, people can extract the sound structure of speech well before they can talk; even newborns do so^25^.

This analysis raises the hypothesis that the two ingredients of language—categorization and combination—are *different* functions in the human brains. While categorization relies on simulation that engages the speech motor system of the brain, the combinatorial process (i.e., phonology) is abstract, and it engages Broca’s area.

Here we explore this hypothesis using neuronavigated transcranial magnetic stimulation (TMS). In two TMS experiments, we examined the causal role of two left hemisphere (LH) areas—the lip motor area (motor representation of the orbicularis oris muscle, OO) and Broca’s area (specifically, the Pars Triangularis, PT, of the frontal operculum)—in two linguistic functions: speech categorization and combination.

To gauge the categorization function, we asked participants to identify ambiguous speech sounds, as in past TMS research^7–13^. Previous TMS research, however, confounded the stimulated articulator with response. This confound arose because the two ambiguous sounds (e.g., *ba* vs. *da*) were produced by two different articulators (e.g., lips vs. tongue), and the stimulated articulator (e.g., lips) was congruent with only one of the two options (e.g., *ba*)^7–13^. Accordingly, the effect of stimulation could have resulted from the perturbation of response selection, rather than speech perception per se^26,27^.

To dissociate the role of the target articulator in speech perception and response, in the present research, we examined the perception of a voicing contrast (e.g., *ba/pa*). Since the stimulated articulator—the lip—is equally relevant to both response options (to both *ba* and *pa*), the effect of stimulation cannot possibly arise from response selection. Still, if speech perception requires the formation of an articulatory plan that correctly specifies all phonetic features (including voicing), and stimulation disrupts the formation of this plan, then the effect of stimulation ought to vary selectively, depending on the congruence of the stimulated articulator (lips) with the speech sound (*ba/pa* vs. *da/ta*). Such results would suggest that phonetic categorization relies on motor simulation.

To gauge the combinatorial function, we further examined speakers’ sensitivity to the structure of syllables such as *bnif* and *lbif*—structures that do not exist in English (participants’ native language). We targeted unattested syllables (as opposed to attested ones, such as *god/dog*) because the identification of familiar syllables heavily relies on memory (i.e., lexical associations). Unattested syllables, by contrast, require participants to generalize their linguistic knowledge to novel forms, and as such, they present a better opportunity to assess the combinatorial function (which is productive^2,3^). Furthermore, past research has shown that the population of interest—English speakers—both adults^28–35^ and children^34,36–38^—are highly sensitive to the structure of such unattested syllables; in fact, sensitivity to syllable structure is evident in newborns^39^. Accordingly, the investigation of unattested syllables offers both a more specific and feasible test of the combinatorial function.

In both tasks, stimulation was administered using TMS. TMS perturbs neural activity in the targeted cortical areas^40^. By using TMS, we were able to gauge the causal role of each site in each computation of interest—categorization and combination—and assess whether they dissociate in human brains. The use of individual MRI-guided neuronavigation allowed us to precisely target the areas of interest reliably in each participant. Furthermore, in each task, the stimulation (to the OO and PT) was applied to each participant at multiple intervals following the target. By applying stimulation at multiple intervals from the onset of the auditory target, we were able to compare these computations with respect to the time-course of activating these brain sites. Thus, we were able to offer novel insights into the spatial and temporal properties of brain activity that play a causal role in the neural implementation of speech categorization and computation.

Summarizing, then, in two experiments, we compared the effect of stimulating the OO and the PT on two linguistic functions—speech categorization (e.g., *ba/pa*?) and combination (e.g., *bnif* vs. *lbif*; see Fig. 1). Both functions were gauged in the same participants using comparable tasks (binary classification).

Considering phonetic categorization, if the previous reports^7–13^ indeed reflect the effect of stimulation on speech perception (and not merely in response), then perception of labial sounds (e.g., *ba/pa*) ought to rely on the motor presentation of the OO. Consequently, TMS to the OO motor representation should disrupt the perception of labial sounds (e.g., *ba/pa*) more than non-labial, coronal (e.g., *da/ta*) sounds (compared with TMS to the PT). Moving to the combinatorial function, here, past results suggest that the perceiving the structure of unattested syllables may be impervious to motor stimulation of the OO^22^, and instead, it recruits the PT^24^. Accordingly, TMS-induced disruption of the PT should reduce sensitivity to phonological combinations more than TMS to the OO.

In Experiment 1 (categorization), each block of trials repeatedly presented a single ambiguous sound, and participants were asked to categorize that sound (e.g., *ba* or *pa*?; as in^16^). To further determine whether the effect of stimulation is due to the role of the relevant articulator (lips) in speech perception or response, we compared these variables across two blocks. One block featured a sound that was ambiguous with respect to its place of articulation (between *ba/da*); in this block, the lips are only congruent with one of the two competing responses (*ba*). In two other blocks, the sound was ambiguous with respect to its voicing—either a labial (e.g., *ba/pa*) or coronal (*da/ta*) sound, such that the lips are dis-confounded with response, as the response options are either both labials (*ba/pa*) or not (e.g., *da/ta*).

If the motor system mediates response selection, then OO stimulation ought to affect performance only when the lips are confounded with response. But if motor simulation mediates speech perception, then disruption of the OO motor representation should affect perception even when the OO is irrelevant to response. If so, then OO stimulation ought to disrupt the perception of voicing for the ambiguous labial sound (to *ba/pa*) more than the ambiguous coronal (*da/ta*) sounds.

**Figure 2:**
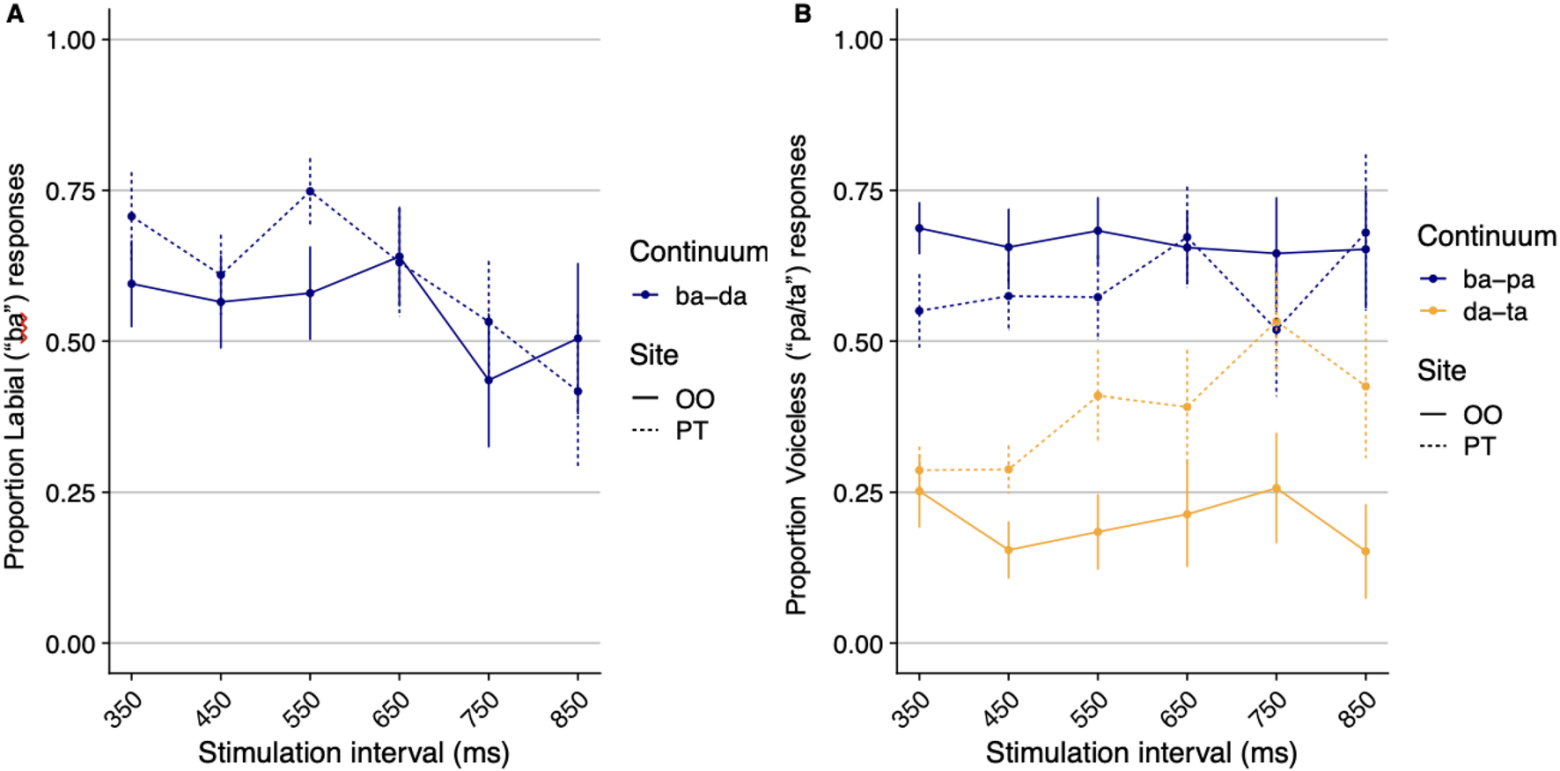
The effect of stimulation site (OO/PT) on the categorization of place of articulation (ba/da; **A**) and voicing (**B**). In the *ba-pa* continuum; *ba* is the voiced response (*pa* is voiceless); In the *da-ta* continuum; *da* is the voiced response (and *ta* is voiceless). Error bars are SEM.

Results (Fig. 2) showed that perception of sounds that were ambiguous with respect to their place of articulation (in between *ba/da*) was not significantly affected by stimulation Site (*Z* < 1), nor did Site interact with the stimulation Interval (*β* = −0.19, *SE* = 0.15, *Z* = −1.27, *p* = 0.21; see also Supplementary Material; SM). In contrast, stimulation selectively modulated the perception of voicing, as Site interacted with Continuum (*ba/pa* vs. *da/ta*: *β* = −1.52, *SE* = 0.47, *Z* = −3.24, *p* = 0.001).

A test of the simple effect of Site showed that the stimulation of the OO had opposite effects on labial and coronal sounds. For the coronal (*da/ta*) sound, OO stimulation increased “voiced” (i.e., *da*) response (relative to the PT, *β* = 1.37, *SE* = 0.36, *Z* = 3.760, *p* < 0.001). For the labial sound, by contrast, OO stimulation tended to attenuate “voiced” (i.e., *ba*) responses (relative to the PT, *β* = −0.32, *SE* = 0.19, *Z* = −1.69, *p* = 0.09).

Thus, Experiment 1 is the first TMS study to demonstrate a causal role of motor simulation even when the targeted area (the lips) is dissociated from response selection (as the lips are equally relevant to both responses, *ba* and *pa*), and even when the role of the articulatory motor system is compared to the role of the PT—a linguistically-relevant area (rather than other motor regions, such as the hand motor area).

The finding that TMS to the OO affected the perception of *voicing*—a feature that is controlled by the larynx (rather than the lips), could suggest that, to perceive a given feature (e.g., voicing, in *ba*), listeners must simulate the articulatory plan of that sound *as a whole.* Disruptions of that plan by TMS to OO affects *all* that sound’s features (including those that are not directly controlled by the lips). And since labial and coronal sounds differentially engage the OO, TMS to the OO produced opposite effects on the perception of voicing in labials and coronals. Motor simulation, then, mediates the categorization of speech sounds.

Experiment 2 examined the brain network that supports the extraction of combinatorial structure of language in the perception of syllables that are unattested in English, their native language. These monosyllables featured consonant-combinations of two types—stop-nasal combinations (e.g., *bnif*) or sonorant-stop combinations (e.g., *lbif*). Participants heard these monosyllables along with their disyllabic counterparts (e.g., *benif* vs. *lebif*), and indicated the number of syllables (one/two).

Past research has shown that the computation of syllable structure engages the PT^24^. Thus, ill-formed monosyllables activate the PT more than better-formed ones (e.g., *lbif* > *bnif*), and monosyllables activate the PT more than disyllables, as disyllables, such as *benif/lebif,* are better-formed compared to their unattested monosyllables counterparts). Of interest is whether the role of the PT in these combinatorial computations is *causal.* If it is, then sensitivity to syllable structure ought to diminish by disrupting the PT (relative to OO). In addition, several studies have suggested that the linguistic role of the PT varies by gender (e.g.^41–43^). Therefore, if males and females differ with respect to their reliance on the left PT^41^, then the effect of PT stimulation could vary by gender.

**Figure 3:**
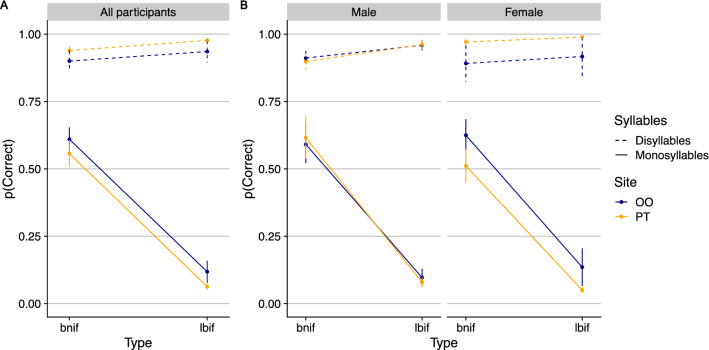
The effect of stimulation on syllable count across participants (**A**) and by gender (**B**). Error bars are SEM. P(Correct) is the proportion of correct responses.

The mixed effect model (see SM) yielded a reliable interaction of Syllable x Type X Site (see Fig. 3A; *β* = 0.68, *SE* = 0.29, *Z* = 2.36, *p* = 0.02). We interrogated this interaction using paired comparisons as executed in the *emmeans* package, using the Tukey method to adjust alpha to control for family-wise error rate.

As expected, well-formed monosyllables (e.g., *bnif*) yielded higher accuracy than ill-formed monosyllables (e.g., *lbif*), and this was the case regardless of whether the stimulation targeted the OO (*β* = −3.13, *SE* = 0.37, *Z* = −8.518, *p* < 0.0001), or the PT (*β* = −3.61, *SE* = 0.37, *Z* = −9.66, *p* < 0.0001). Additionally, disyllables elicited more accurate responses than monosyllables, and this was the case regardless of syllable Type and stimulation Site (for the OO and PT: *p* < 0.0001). Responses to the two types of disyllables (*benif* vs. *lebif*), however, did not differ (for both Sites: Z < 1.1).

However, response accuracy was systematically modulated by stimulation Site. For the monosyllables, the stimulation of the PT systematically attenuated response accuracy to the ill-formed syllable type (e.g., *lbif; β* = 0.72, *SE* = 0.17, *Z* = 4.29, *p* < 0.001), but not the well-formed *bnif*-type (*β* = 0.23, *SE* = 0.13, *Z* = 1.83, *p* > 0.60). For disyllables, PT stimulation improved accuracy compared to OO stimulation (for *benif: β* = −1.51, *SE* = 0.20, *Z* = −7.44, *p* < 0.0001; for *lebif*: *β* = −1.71, *SE* = 0.24, *Z* = −7.29, *p* < 0.0001).

The effect of stimulation Site further interacted with Gender (Fig. 3B), yielding a significant Gender × Site × Syllable interaction (*β* = −4.26, *SE* = 0.31, *Z* = −13.57, *p* < 0.001).

Paired comparison tests indicated that the stimulation Site had no effect on males (both Z < 1). In females, however, the stimulation of the PT (compared to OO stimulation) promoted disyllabic responses, regardless of whether the target was, in fact, disyllabic (i.e., an increase in correct responses: *β* = −3.24, *SE* = 0.29, *Z* = −11.27, *p* < 0.0001) or monosyllabic (i.e., an increase in false alarms: *β* = 0.98, *SE* = 0.17, *Z* = 5.65, *p* < 0.0001). Accordingly, females were less accurate than males only when TMS targeted the PT and the stimuli were disyllabic (*β* = 3.57, *SE* = 1.03, *Z* = 3.45, *p* < 0.02). There was no gender difference in responses to monosyllables, regardless of whether TMS targeted the OO (Z < 1) or PT (*β* = −0.65, *SE* = 0.328, *Z* = −1.98, *p* > 0.49). There was also no gender difference in response to disyllables when TMS targeted the OO (*Z* < *1*). A signal-detection analysis confirmed that PT stimulation produced in females a highly-selective response bias (*β*; see SM)—it emerged only for disyllables (not monosyllables), and only when stimulation targeted the PT (but not OO). Furthermore, the two genders did not differ in speech categorization (in Experiment 1, Z < 1).

The exquisite selectivity of this pattern makes it clear that female participants were not uniformly biased. Rather, they only showed bias when structure-building was disrupted. The fact that this bias was only elicited by PT stimulation further suggests that the left PT plays a causal role in the computation of combinatorial structure in these female participants. Consequently, when the PT is disrupted, structure-sensitivity declines, and in its place, response bias emerges.

The finding that males were largely impervious to stimulation further suggests that, unlike females, in male participants, structure-building is less reliant on the left PT. One possibility is that these males built structure by engaging the right PT. This would be consistent with past research^41^, suggesting that long-range functional connectivity in frontal areas is less lateralized in male brains. Alternatively, these male participants might have extracted combinatorial structure by relying on other brain structures in either hemisphere. Either way, however, gender seems to have influenced the likelihood of engagement of the left PT in language structure building.

The results of this single study with a small sample size cannot settle the longstanding debate on gender differences in language. However, our results suggest it ought to be reformulated. If language comprises of distinct neural functions, then the question of dimorphism in “*the* language network” might be too coarse to settle. Instead, we advocate a granular, functionally-informed approach that compares males and females in *specific* linguistic computations (e.g., combination).

Two methodological limitations of our study ought to be noted. First, our study may not have been able to adequately assess the effect of stimulation interval. This was the case, as at the longer stimulation intervals, many responses were given prior to the onset of stimulation, so these data were removed from the analysis (see SM, and Tables S2 and S6). The loss of data limited our assessment of stimulation chronometry. Second, given that our TMS procedure identified the PT via a structural MRI, and that the functional localization of Broca’s area varies^44^, it is conceivable that the localization of the PT was imprecise. These limitations notwithstanding, our results suggest that the OO and the PT differ in their contributions to speech perception.

**Figure 4:**
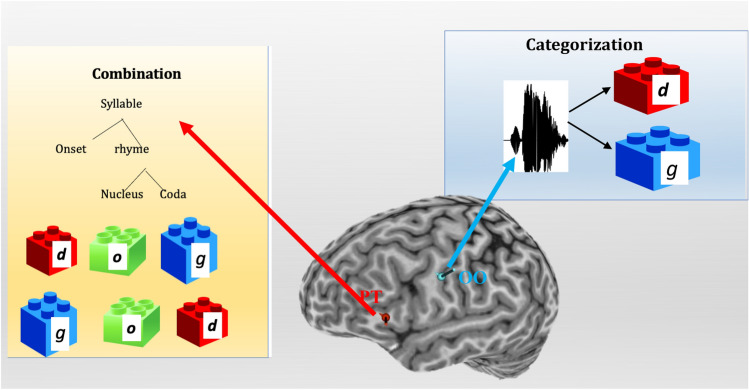
Graphical summary. Our results suggest that the categorization of speech sounds engages the motor, whereas the combinatorial computation of syllable structure engages the PT.

This is the first study to demonstrate that speech categorization and combination differ with respect to their neural implementation (see Fig. 4). Speech categorization engages the articulatory motor system, possibly because participants attempt to simulate the production of those sounds by engaging the relevant articulator (e.g., lips, for labials). Moreover, our study shows for the first time that the effect of stimulation obtains even when the stimulated articulator (the lips) is dissociated from response. The perception of speech categories thus recapitulates their articulation.

The computation of combinatorial structure, by contrast, is not dependent on motor simulation, and instead, recruits the left PT. Moreover, our results tentatively suggest that the contribution of PT to the computation of syllable structure (but not to phonetic categorization) might differ for males and females.

We thus conclude that the two ingredients of language—categorization and combination—are distinct functions in the human brain. Language, then, is preferentially a vocal system of communication, but the human capacity for language goes far beyond the vocal command of speech.

## Methods

### Participants

Sixteen participants took part in this experiment (9 females, 7 males) ^9females,7males^. Data from the first (female) participant was incomplete, due to a technical malfunction of the TMS equipment (for additional characteristics, see SM). Sample size was informed by the previous TMS literature on phonetic categorization^7–13^, as well as by analysis of our previous TMS study^22^, suggesting that the selected sample size is sufficient to detect the effects of stimulation on the computation of syllable structure at the with a power of 0.8 at the alpha level of 0.05.

The protocols for Experiments 1–2 were reviewed and approved by the Institutional Review Board of Beth Israel Medical Center and Northeastern University, and the experiments were administered in adherence of those protocols. All participants in Experiments 1–2 signed a written informed consent prior to taking part in the experiment.

### Materials

The materials consisted of three ambiguous speech sounds. One sound was ambiguous with respect to its place of articulation (in between *ba/da*); the two other sounds were ambiguous with respect to their voicing—one labial (in between *ba/pa*), another coronal (in between *da/ta*). These three stimuli corresponded to the perceptual midpoint of a 10-step continuum, used in past research^16^. The perceptual midpoint was calculated for each individual participant. These calculations and the preparation of the ambiguous midpoint stimuli are detailed in the SM.

### Design

Within each stimulation site, each of the three ambiguous stimuli (*ba/da, ba/pa* and *da/ta*) was presented in three distinct block trials. Each such block, in turn, repeatedly presented a single stimulus at all seven TMS stimulation intervals equally (with order randomized).

Participants performed the categorization task twice, on two separate sessions (separated by a minimum of six days). In one day, the stimulation was applied to the PT, in another, stimulation was applied to the OO, with order balanced as closely as possible across participants.

Within each day, participants were always presented with the combinatorial (phonological) task first, followed by the categorization (phonetic) task. We deliberately presented the syllable count second, as past research has shown that promoting attention to phonetic detail (e.g., in the phonetic task) systematically alters performance on the syllable count task (whereas there are no known reverse cross-overs, from phonology to phonetics)^33^.

### Transcranial magnetic stimulation

TMS targets within the OO and PT were identified anatomically on the Montreal Neurological Institute (MNI) template brain and nonlinearly transformed into subject space using FSL (FMRIB Group; Oxford, UK). The OO target was further refined during a prior visit as the location where the largest and most consistent MEP could be elicited from the right OO muscle with single pulse TMS (for additional details, see SM).

### Procedure

Participants were told that, in each trial, they would hear a syllable and asked to identify it (e.g., as *ba* or *pa*). They were advised to pay close attention, as the syllables are ambiguous, and they are followed by white noise. Participants were asked to respond as fast and accurately as possible.

The three stimuli (*ba/da, ba/pa, da/ta*) were presented in three blocks (with order counterbalanced). Prior to each block, participants were briefly informed of the two sounds they were about to hear (e.g., *ba or* pa?) and the task was described. Each such stimulus was administered in mini-blocks of 28 trials (balanced for simulation interval), and participants were encouraged to take breaks between mini-blocks. Within block, trial order was randomized.

Each trial began with the presentation of a fixation point (*) for 200 ms, followed by the ambiguous auditory stimulus, presented for 2700 ms; the next trial followed automatically. The onset of the auditory stimulus triggered the presentation of a train of five TMS pulses, presented 20 ms apart (at 50 Hz). The interval between the onset of the stimulus and the onset of the TMS stimulation was either 350 ms, 450 ms, 550 ms, 650 ms, 750 ms, 850 ms or 950 ms (7 intervals total).

## Experiment 2

### Participants

Sixteen participants took part in the experiment (these participants also took part in Experiment 1). As noted in Experiment 1, data from the first participant was incomplete due to technical malfunction.

### Materials

The materials consisted of 28 matched pairs of monosyllabic words (e.g., *bnif, lbif*), along with their disyllabic counterparts (e.g., *benif; lebif)*; these items were employed in past research^22,29^, and they are provided in Supplementary Appendix).

All monosyllables began with a consonant combination that is unattested in English, either a stop-nasal (e.g., *bnif*) or sonorant-stop (e.g., *lbif*); the matched disyllables were identical, except that the two initial consonants were separated by a schwa (e.g., *benif* vs. *lebif*). These materials were recorded by a native Russian speaker; since these consonant combinations are all attested in Russian, the speaker was able to produce all items naturally. And indeed, past research has confirmed that Russian participants do perceive these items as intended by the speaker (as monosyllables or disyllables)^28^.

Each such stimulus in Experiment 2 was edited precisely as described in Experiment 1. Thus, for each stimulus, we first removed all silences. We next inserted a silent interval of 800 ms at the end of stimulus, followed by an interval of white noise, such that the entire stimulus duration was 2700 ms.

### Design

Each participant was presented with all 112 words (28 items × 2 types × 2 syllables) in all 7 stimulation intervals, for a total of 784 trials per Simulation site. As in Experiment 1, we divided the trials into mini-blocks of 28 trials each, such that (a) each mini-block was balanced for the 2 Type × 2 Syllable × 7 Interval conditions, and (b) each individual item was presented to each participant once with each of the 7 stimulation intervals. The order of the mini-blocks was varied across participants, and it was further matched for the OO and PT conditions (for one participant, the order differed due to an experimenter error). Critically, within each mini-block, the order of the 28 trials (i.e., the ordering of the simulation intervals and items) was randomized.

### Procedure

As in Experiment 1, each participant was administered the task twice, once with OO stimulation and once with PT stimulation (as described in Experiment 1). Within each such session, participants were first given the syllable count task (Experiment 2) followed by the categorization task (Experiment 1).

Participants were told that, in each trial, they would hear words spoken by a foreign speaker; their task was to decide if the word has one syllable or two and respond by pressing one of two keys. Participants were first given practice with existing English words (e.g., *sport* vs. *support*), followed by the experimental session.

The experimental session was delivered in mini-blocks of 28 trials (balanced for syllable × type × interval), presented randomly. Participants were encouraged to take breaks between mini-blocks.

The structure of the trial was as in Experiment 1. Thus, each trial began with a fixation (*) displayed for 200 ms, followed by the auditory stimulus (lasting 2700 ms); the next trial followed automatically.

The onset of the auditory stimulus triggered the presentation of a train of six TMS pulses, presented 20 ms apart (50 Hz). The interval between the onset of the stimulus and the onset of the TMS stimulation was either 350 ms, 450 ms, 550 ms, 650 ms, 750 ms, 850 ms or 950 ms (7 intervals total). The TMS procedure was exactly as in Experiment 1 (for additional information, see SM).

## Supplementary Information

Supplementary Information.

## Data Availability

All data are available as supplementary materials (10.1038/s41598-023-28099-w).
