# Supplementary material for: Phonetic categorization relies on motor simulation, but combinatorial phonological computations are abstract
Source: Sci Rep. 2023 Jan 17;13:874. doi: 10.1038/s41598-023-28099-w (PMC9845317; doi:10.1038/s41598-023-28099-w)
Supplement: Supplementary file 1 — Supplementary Information. [file 41598_2023_28099_MOESM1_ESM.docx]

**Phonetic categorization relies on motor simulation, but combinatorial phonological computations are abstract**

***Supplementary Materials:***

**Authors:** Iris Berent^1^*, Peter J. Fried^2,3^, Rachel M. Theodore^4^, Daniel Manning^2^, Alvaro Pascual-Leone ^3,5,6^

**Affiliations:**

^1^ Department of Psychology, Northeastern University, Boston, Massachusetts, USA

^2^ Berenson-Allen Center for Noninvasive Brain Stimulation, Beth Israel Deaconess Medical Center, Boston, Massachusetts, USA

^3^ Department of Neurology, Harvard Medical School, Boston, Massachusetts, USA

^4^ Department of Speech, Language, and Hearing Sciences, University of Connecticut

^5^Hinda and Arthur Marcus Institute for Aging Research and Deanna and Sidney Center for Memory Health, Hebrew SeniorLife, Boston, Massachusetts, USA

^6^ Guttmann Brain Health Institute, Barcelona, Spain

* Corresponding author. Email: i.berent@northeastern.edu

**Experiment 1**

**Methods**

***Participants*.** Participants were young adults (M=21.9 years, SD=3.83), monolinguals, and native speakers of English. Native English speakers were defined as individuals who have acquired English before the age of five. Monolinguals were defined as participants who have not acquired a second language (other than English) before the age of 10 and spoke that second language fluently at home.

In addition, we excluded participants who reported having language or reading disorders, neurologic disorders, major psychiatric illnesses (well-controlled depression or anxiety was permitted on a case-by-case basic following review by a psychiatrist), uncontrolled medical conditions, substance abuse or dependence within the previous 6 months, history of epilepsy, seizures, or fainting spells of unknown origin, metal in the brain or skull (other than dental work) or implanted medical devices.

***Materials****.* The materials consisted of three ambiguous speech sounds (*ba/da; ba/pa, da/ta*)*.* We next describe the generation of the continua, and the procedure used to estimate its perceptual midpoint.

*The generation of the continua.* The continua were generated from recordings, generated by a female, native speaker of English*.* Stimulus manipulations were performed using SIGNAL software (Engineering Design, Berkeley, CA) and Matlab (Mathworks, Natick, MA). All continua were made from recordings of isolated syllables produced by native speakers (16 bits, 44.1 kHz sampling rate).

The *ba-pa* continuum was produced by removing the DC component from both endpoint syllables, setting non-vocalization portions of the recording to zero, and truncating the recording lengths to the shorter of the two stimuli. The *ba* syllable was rotated (zero-value segments added/subtracted at the beginning and end, while keeping the file length the same) so that its vowel periodicity aligned with the *pa* syllable, and a ‘hybrid *pa*’ syllable was created using the first 159.19 ms of the *pa* and the rest of the *ba* from 159.19 ms to the recording end, joined at a zero-crossing. The noisy initial part (the aspiration phase) of this ‘hybrid *pa*’ syllable was then progressively shortened at successive zero crossings occurring within the time interval between 114-131 ms to make the eight steps of the continuum. The ‘*pa*’ endpoint had a value of 57.8 ms from the start of articulation to the end of the aspiration phase; this value was 37.1 ms for the ‘*ba*’ endpoint.

The *da-ta* continuum was produced by removing the DC component from both endpoint syllables, setting non-vocalization portions of the recording to zero, and truncating the recording lengths to the shorter of the two stimuli. The *da* syllable was then rotated to align its vowel periodicity with the *ta* syllable, and a ‘hybrid *ta*’ syllable was created using the first 183.38 ms of the *ta* and the rest of the *da* from 183.38 ms to the recording end. The first 6 stimuli in the continuum were made by progressively shortening the noisy part of the ‘hybrid *ta*’ syllable starting from the zero crossing that was proximal to the start of the voicing for the vowel and proceeding backwards at successive zero crossings occurring every 7-10 ms. The rest of the stimuli in the continuum were made by successively replacing the remaining portion of the noisy signal before the start of the voicing by successive voiced vowel periods present in the original *da* syllable at these same positions in time (splicing done at zero crossings), ending up with a perfect reproduction of the original *da* syllable at the end of the continuum.

The *ba/da* continuum was generated using a custom-written Praat script (*1*). We generated the continua between two voiced speech sounds by first using the pitch-synchronous overlap and add (PSOLA) technique to equate their durations and pitch contours, and then by interpolating between the two sounds in steps of 0.1 to produce 10-step continua.

*Midpoint estimation.* To estimate the perceptual midpoint of the continuum, each participant took part in a calibration session, presented in a separate visit prior to the main experiment. In that session, each participant was presented 20 times with all ten steps of each continuum (with order randomized), delivered in three counterbalanced blocks (one block per continuum).

We calculated the perceptual midpoint as follows. For each participant, the quickpsy package in R (*2*) was used to fit the identification responses to a cumulative normal function, constraining the guess and lapse rates to be within 5% following procedures for fitting psychometric functions (e.g., (*3, 4*)), with the mean of the derived function representing the perceptual midpoint. Across participants, the selected midpoints for all steps corresponded to step 6. Specifically, for the *ba/pa* contrast: M=6.18 (SD=0.54); for the *da/ta*: M=6.23 (SD=0.41); for *ba/da:* M=6.03 (SD=0.68).

*The preparation of the ambiguous stimulus (midpoint).* After we had identified the perceptual midpoint, we edited that stimulus (in Praat; (*1*)) to remove any silences. To render the repetition of the stimulus less obvious, we next inserted a silent interval of 800 ms at the end of the sound, followed by white noise (at 68dB), in Praat (*1*)) using a custom-made script. The length of the white noise interval varied, so that the entire stimulus duration (including silence) was 2700 ms.

*Design.* Within each stimulation site, each of the three ambiguous stimuli (*ba/da, ba/pa* and *da/ta*) was presented in three distinct block trials. Each such block, in turn, repeatedly presented a single stimulus at all seven TMS stimulation intervals equally (with order randomized). For most participants (14/16), the ambiguous stimulus was repeated 20 times per interval, for a total of 840 trials (3 stimuli x 20 repetitions x 7 intervals x 2 sites). For the initial two participants, the number of repetitions was doubled (1680 trials total); because this lengthy session was not easily tolerated, we reduced the number of trials for the third participant on.

Participants performed the categorization task twice, on two separate sessions (separated by a minimum of six days). In one day, the stimulation was applied to the Pars Triangularis (PT), in another, stimulation was applied to the Orbicularis Oris (OO), with order balanced as closely as possible across participants. Thus, of the nine females, five received the OO stimulation first; of the seven males, four received the OO stimulation first.

***Transcranial Magnetic Stimulation.*** All parameters used in the study conformed to current recommended guidelines for the safe application of TMS endorsed by the International Federation of Clinical Neurophysiology (IFCN) (*5*). Motor evoked potential (MEPs) elicited by single-pulse TMS were recorded from right first dorsal interosseous (FDI) and OO muscles using surface electromyography. The hotspot, corresponding to the location within the motor cortex that elicited the largest and most consistent MEPs, was determined for the right FDI followed by the right OO during the first TMS visit. Following IFCN guidelines (*6*), each individual’s resting motor threshold (RMT) were measured from the right FDI muscle with PowerLab amplifier and LabChart software (ADInstruments, USA). The RMT FDI was used to set the intensity of subsequent stimulation for both OO and PT sites. FDI was selected over OO because (1) it is a significantly more widely used region in TMS studies, allowing stimulation intensities to be better compared to other research, and (2) MEPs from the OO region are not as consistently elicited at lower intensities, which made estimation of RMT less reliable and stable than for FDI. TMS was administered using a cooled figure-of-8 coils connected to a MagPro X100 stimulator (MagVenture A/S, Denmark), and used to set the intensity of subsequent stimulation. The neuronavigation system Brainsight® (Rogue Research, Canada) was used to identify the stimulation targets and ensure consistent targeting throughout and across the experimental sessions.

For the OO and PT targets, we first identified the regions anatomically on the Montreal Neurological Institute (MNI) template brain. For OO, we selected coordinates (-59, -5, 39) based on a prior TMS study (*7*) For PT, we selected coordinates (-50.0, 31.3, 8.2) corresponding to the crest of the gyrus adjacent to the inferior frontal sulcus. We used a custom processing script in BASH to take each subject’s anatomical MRI, create a non-linear transform from the subject’s native space to MNI space and then use the inverse of that transform to bring the coordinates into subject’s space using freely available tools from the FMRIB Software Library (FSL; Oxford, UK; [https://fsl.fmrib.ox.ac.uk/fsl/](https://nam12.safelinks.protection.outlook.com/?url=https%3A%2F%2Ffsl.fmrib.ox.ac.uk%2Ffsl%2F&data=05%7C01%7CI.Berent%40northeastern.edu%7C46c7432c7a1148208f1308da4960fed5%7Ca8eec281aaa34daeac9b9a398b9215e7%7C0%7C0%7C637902978190665324%7CUnknown%7CTWFpbGZsb3d8eyJWIjoiMC4wLjAwMDAiLCJQIjoiV2luMzIiLCJBTiI6Ik1haWwiLCJXVCI6Mn0%3D%7C3000%7C%7C%7C&sdata=ijfdwfkXJTOc5OZyAc7bh0zfJcL0TSH%2BJm56fGnRObI%3D&reserved=0)). The OO target was further refined physiologically as described above.

Table S1 provides the stimulation intensity for males and females in the two sites. The gender differences did not approach significance (all t<1).

**Table S1. Mean stimulation intensity in Experiment 1**

|  |  | ***OO*** | ***PT*** |
| --- | --- | --- | --- |
| **Female** | *Mean* | 34.89 | 37.00 |
|  | *SD* | 6.09 | 6.34 |
| **Male** | *Mean* | 34.71 | 34.86 |
|  | *SD* | 7.68 | 7.00 |
|  | t(11) | 0.39 | 0.30 |
|  | *p* | 0.71 | 0.77 |

***Analysis*.** We limited to the analysis to responses occurring after the onset of stimulation (e.g., for the 350 ms interval, we excluded response times shorter than 350 ms). Because, in the last interval (950 ms), most responses (89%) were delivered prior to the onset of stimulation, this interval was eliminated from all analyses. Table S2 provides the percentage of responses that were excluded from the analysis of Experiment 1. As expected, the exclusion affected mainly the longer intervals, but it was comparable for the two stimulation sites.

**Table S2. The percentage of responses excluded from the analysis of Experiment 1.**

| Continuum | BaDa | | PaBa | | TaDa | |
| --- | --- | --- | --- | --- | --- | --- |
| interval/Site | OO | PT | OO | PT | OO | PT |
| 350 | 22 | 15 | 18 | 8 | 23 | 17 |
| 450 | 41 | 36 | 32 | 23 | 42 | 34 |
| 550 | 62 | 54 | 48 | 47 | 55 | 52 |
| 650 | 75 | 69 | 61 | 64 | 75 | 67 |
| 750 | 84 | 79 | 73 | 81 | 84 | 78 |
| 850 | 86 | 90 | 81 | 90 | 85 | 86 |
| 950 | 89 | 92 | 84 | 92 | 89 | 91 |

**Results**

Participants’ binary responses were submitted to a mixed effects logistic regression model with (Voiceless ~ Continuum.c * Site.c * Interval.s * Gender + (Continuum.c * Site.c * Interval.s |Subject). In this and all subsequent logistic regression models, significance is evaluated using Wald tests.

*Place of articulation*. In addition to the results described in the main text, the model also yielded a marginally significant interaction of Site x Gender (*β*=-1.95, *SE*=1.04, *Z*=-1.88, *p*=.06). However, Tukey HSD tests found no significant difference between the means (all p’s >.22). The effect of Interval.s was nearly significant (*β*=-0.199, *SE*=-.10, *Z*=-1.96, *p*=.05), as the proportion of “*ba*” response tended to decrease at longer stimulation intervals. The full model output is pvodied in Table S3.

**Table S3 The output of the logistic regression model of Experiment 1 for *place of articulation***

|  |  | *Estimate* | *SE* | *z* | *p* |
| --- | --- | --- | --- | --- | --- |
| (Intercept) |  | 0.5094 | 0.2165 | 2.353 | 0.0186 |
| Site1 |  | 0.4945 | 0.5127 | 0.965 | 0.3348 |
| **Interval.z* |  | *-0.198* | *0.1009* | *-1.961* | *0.0498* |
| gender1 |  | 0.3261 | 0.4361 | 0.748 | 0.4546 |
| Site1:Interval.z |  | -0.1864 | 0.1471 | -1.267 | 0.2052 |
| Site1:gender1 |  | -1.9462 | 1.0371 | -1.877 | 0.0606 |
| Interval.z:gender1 |  | -0.2404 | 0.2043 | -1.177 | 0.2392 |
| Site1:Interval.z:gender1 |  | -0.1268 | 0.3141 | -0.404 | 0.6864 |

*Voicing contrast.* In addition to the results described in the main text, the model also yielded significant main effects of Continuum (*β*=2.10, *SE*=0.45, *Z*=4.68, *p*<.001) and Site (*β*=0.48, *SE*=0.20, *Z*=2.38, *p=.*02), as voiceless responses were more likely in the labial (*ba-pa*) relative to the coronal (*da-ta*) continuum, and when stimulation targeted the PT relative to the OO.

**Table S4 The output of the logistic regression model of Experiment 1 for the *voicing* contrast.**

|  | *Estimate* | *SE* | *z* | *p* |
| --- | --- | --- | --- | --- |
| (Intercept) | -0.40884 | 0.15689 | -2.606 | 0.00916 |
| **Continuum1* | *2.10325* | *0.44978* | *4.676* | *2.92E-06* |
| *Site1 | 0.47959 | 0.20119 | 2.384 | 0.01714 |
| Interval.z | 0.04033 | 0.066 | 0.611 | 0.54118 |
| gender1 | -0.25361 | 0.30071 | -0.843 | 0.39901 |
| **Continuum1:Site1* | *-1.52003* | *0.46981* | *-3.235* | *0.00121* |
| Continuum1:Interval.z | -0.11494 | 0.13718 | -0.838 | 0.40212 |
| Site1:Interval.z | 0.01824 | 0.15036 | 0.121 | 0.90346 |
| Continuum1:gender1 | 0.30145 | 0.88866 | 0.339 | 0.73445 |
| Site1:gender1 | -0.03977 | 0.36889 | -0.108 | 0.91414 |
| Interval.z:gender1 | 0.16208 | 0.11246 | 1.441 | 0.14951 |
| Continuum1:Site1:Interval.z | 0.03622 | 0.2852 | 0.127 | 0.89895 |
| Continuum1:Site1:gender1 | -0.19427 | 0.87681 | -0.222 | 0.82465 |
| **Continuum1:Interval.z:gender1* | *-0.52571* | *0.2364* | *-2.224* | *0.02616* |
| Site1:Interval.z:gender1 | -0.04871 | 0.27578 | -0.177 | 0.85981 |
| Continuum1:Site1:Interval.z:gender1 | 0.33502 | 0.50223 | 0.667 | 0.50473 |

The only other significant effect was an interaction of Continuum x Interval x Gender (*β*=-0.52, *SE*=0.24, *Z*=-2.22, *p*=.03). In both genders, “voiceless” responses were more likely for the labial (*ba-pa*) relative to the coronal (*da-ta*) continuum. In females, however, this difference was maximal at the short stimulation intervals, whereas males showed the opposite trend. Critically, these effects did not further interact with the stimulation Site (for the Site x Continuum x Interval x Gender interaction, Z<1). Table S4 provides the full model output.

**Experiment 2**

***Materials.*** Table S5 provides the intensity levels per gender. The two genders did not differ significantly.

**Table S5. Mean stimulation intensity in Experiment 1**

|  |  | **OO** | **PT** |
| --- | --- | --- | --- |
| **Female** | *Mean* | 34.89 | 37.00 |
|  | *SD* | 6.09 | 6.34 |
| **Male** | *Mean* | 34.71 | 34.86 |
|  | *SD* | 7.68 | 7.00 |
|  | *t(11)* | 0.71 | 0.77 |
|  | *p* | 0.39 | 0.30 |

As in Experiment 1, performance on the last stimulation interval (950 ms) was excluded from all analysis, as most responses (61%) were given prior to the stimulation onset. Table S6 presents the percentage of excluded responses all intervals. As expected, the exclusion affected primarily the longer intervals, but it was comparable for the two stimulation sites. An additional analysis confirmed that the exclusion of the last interval (950 ms) did not affect the conclusions.

**Table S6. The percentage of responses excluded from the analysis of Experiment 2**

| **Type** | **Syllables** | **Interval** | **OO** | **PT** |
| --- | --- | --- | --- | --- |
| *bnif* | *one* | 350 | 0 | 0 |
|  |  | 450 | 1 | 1 |
|  |  | 550 | 3 | 7 |
|  |  | 650 | 14 | 15 |
|  |  | 750 | 29 | 32 |
|  |  | 850 | 47 | 49 |
|  |  | 950 | 63 | 63 |
|  | *two* | 350 | 0 | 1 |
|  |  | 450 | 1 | 1 |
|  |  | 550 | 4 | 7 |
|  |  | 650 | 16 | 16 |
|  |  | 750 | 30 | 31 |
|  |  | 850 | 46 | 48 |
|  |  | 950 | 59 | 62 |
| *lbif* | *one* | 350 | 1 | 0 |
|  |  | 450 | 1 | 0 |
|  |  | 550 | 4 | 6 |
|  |  | 650 | 13 | 14 |
|  |  | 750 | 29 | 31 |
|  |  | 850 | 48 | 46 |
|  |  | 950 | 58 | 60 |
|  | *two* | 350 | 1 | 0 |
|  |  | 450 | 0 | 1 |
|  |  | 550 | 2 | 3 |
|  |  | 650 | 12 | 15 |
|  |  | 750 | 27 | 29 |
|  |  | 850 | 46 | 48 |
|  |  | 950 | 64 | 58 |

**Results**

Response accuracy was submitted to a mixed effect logistic regression model with random intercepts by participants and random slopes by participant for site, syllable, type and interval, and item slopes by type (accuracy~ gender*interval.s*site*syllable*type + (interval.s+site+syllable+type|subject) + (type|pair)). Table S7 provides the full model output.

**Table S7 The output of the logistic regression model for Experiment 2**

|  | ***Estimate*** | ***Std. Error*** | ***Z*** | ***p*** |
| --- | --- | --- | --- | --- |
| (Intercept) | 1.593509 | 0.213204 | 7.474 | 0.0000000 |
| **gender1* | *0.895635* | *0.346187* | *2.587* | *0.0096800* |
| interval.s | 0.002767 | 0.042763 | 0.065 | 0.9484000 |
| **site1* | *-0.568192* | *0.135875* | *-4.182* | *0.0000289* |
| **syllable1* | *5.508353* | *0.596292* | *9.238* | *< 2e-16* |
| **type1* | *1.524069* | *0.363242* | *4.196* | *0.0000272* |
| gender1:interval.s | -0.078008 | 0.07903 | -0.987 | 0.3236100 |
| **gender1:site1* | *-1.129062* | *0.262623* | *-4.299* | *0.0000171* |
| interval.s:site1 | 0.108296 | 0.069176 | 1.566 | 0.1174600 |
| gender1:syllable1 | 2.089644 | 1.187117 | 1.76 | 0.0783600 |
| **interval.s:syllable1* | *-0.232014* | *0.073355* | *-3.163* | *0.0015600* |
| **site1:syllable1* | *-2.086761* | *0.159746* | *-13.063* | *< 2e-16* |
| gender1:type1 | 0.166005 | 0.458025 | 0.362 | 0.7170300 |
| interval.s:type1 | -0.046636 | 0.070399 | -0.662 | 0.5076800 |
| site1:type1 | -0.14401 | 0.143235 | -1.005 | 0.3147000 |
| **syllable1:type1* | *-3.686314* | *0.155517* | *-23.704* | *< 2e-16* |
| gender1:interval.s:site1 | -0.024404 | 0.137908 | -0.177 | 0.8595400 |
| gender1:interval.s:syllable1 | 0.063169 | 0.143089 | 0.441 | 0.6588700 |
| **gender1:site1:syllable1* | *-4.261107* | *0.313684* | *-13.584* | *< 2e-16* |
| interval.s:site1:syllable1 | 0.189539 | 0.137983 | 1.374 | 0.1695500 |
| gender1:interval.s:type1 | 0.191937 | 0.138335 | 1.387 | 0.1653000 |
| gender1:site1:type1 | -0.205566 | 0.285823 | -0.719 | 0.4720100 |
| interval.s:site1:type1 | -0.100702 | 0.137154 | -0.734 | 0.4628100 |
| gender1:syllable1:type1 | 0.412347 | 0.302311 | 1.364 | 0.1725700 |
| interval.s:syllable1:type1 | 0.060987 | 0.137815 | 0.443 | 0.6581100 |
| **site1:syllable1:type1* | *0.680862* | *0.287976* | *2.364* | *0.0180600* |
| gender1:interval.s:site1:syllable1 | -0.201379 | 0.275551 | -0.731 | 0.4648900 |
| gender1:interval.s:site1:type1 | -0.006175 | 0.274367 | -0.023 | 0.9820400 |
| gender1:interval.s:syllable1:type1 | 0.019222 | 0.274481 | 0.07 | 0.9441700 |
| gender1:site1:syllable1:type1 | 0.053811 | 0.568308 | 0.095 | 0.9245600 |
| interval.s:site1:syllable1:type1 | -0.162328 | 0.274064 | -0.592 | 0.5536500 |
| gender1:interval.s:site1:syllable1:type1 | -0.227182 | 0.547408 | -0.415 | 0.6781300 |

The model yielded significant effects of Gender (*β*=0.89, *SE*=0.35, *Z*=2.59, *p*=.01), Site (*β*=-0.57, *SE*=0.14, *Z*=-4.12, *p*<.001), Syllable (*β*=5.51, *SE*=0.60, *Z*=9.22, *p*<.001) and Type (*β*=1.52, *SE*=0.36, *Z*=4.19, *p*<.001), as females were generally more accurate than males, accuracy was higher when stimulation targeted the OO (relative to the PT), and when the stimuli consisted of disyllables, and for the *bnif*  (relative to the *lbif*) type.

These effects, however, were qualified by the significance of numerous two-way interactions. These interactions were each interpreted using Tukey HSD tests, described below. We note, however, that these two-way interactions ought to be interpreted with caution, due to the higher-order interactions, discussed in the main text.

The Gender x Site interaction (*β*=-1.13, *SE*=0.26, *Z*=-4.29, *p*<.001) indicated that females were more accurate than males only when the stimulation targeted the PT (*β*=1.46, *SE*=0.46, *Z*=3.16, *p*<.01), but not the OO (*β*=0.33, *SE*=0.25, *Z*=1.30, *p*<.56).

The Site x Syllable interaction (*β*=-2.09, *SE*=0.16, *Z*=-13.06, *p*<.001) indicated that the PT attenuated response accuracy to monosyllables (*β*=0.48, *SE*=0.13, *Z*=3.69, *p*<.01), but improved response accuracy to disyllables (*β*=-1.61, *SE*=0.18, *Z*=-8.84, *p*<.001).

The Syllable x Type interaction (*β*=-3.68, *SE*=0.15, *Z*=-23.70, *p*<.001) indicated that *bnif* -type items produced higher accuracy (relative to *lbif*) only for monosyllables (*β*=-3.37, *SE*=0.36, *Z*=-9.28, *p*<.001), but not disyllables (Z<1).

Finally, the Interval x Syllable interaction (*β*=-0.23, *SE*=0.073, *Z*=-3.16, *p*<.01) suggested that the advantage of disyllables was slightly attenuated at longer stimulation intervals. The accuracy results of individual participants are provided in Figure S1.

*Signal detection analysis.* The accuracy results reported in the main text suggest that the stimulation of the PT produced a selective bias in females. To evaluate this possibility, we submitted participants’ bias scores (*β*) to a 2 Gender x 2 Type x 2 Syllable x Site ANOVA*.* The Site X Gender interaction approached significance (F(1,14)=4.01, p=.06, η²_p_=.223).

The simple main effect of stimulation was significant for females (F(1,14)=6.94, p=.03), but not males (F<1). Additionally, the simple main effect of gender approached significance only when stimulation targeted the PT (F(1,14)=3.65, p=.08). There was no hint of gender differences when stimulation targeted the OO (F<1).

A similar ANOVA of sensitivity (d’) found no effects or interactions involving gender or site (all F<1).

**Figure S1.** The performance of individual females (in pink) and male (in blue) participants. Error bars are SE of the means. Mono=monosyllables; di=disyllables.

**References**

1. P. Boersma, D. Weenink. (2018).

2. D. Linares, J. López-Moliner, Quickpsy: An R package to fit psychometric functions for multiple groups. *The R Journal,* **8**, 122-131 (2016).

3. F. Wichmann, N. Hill, The psychometric function: I. Fitting, sampling, and goodness of fit. *Perception & Psychophysics* **63**, 1293-1313 (2001).

4. M. Clayards, M. K. Tanenhaus, R. N. Aslin, R. A. Jacobs, Perception of speech reflects optimal use of probabilistic speech cues. *Cognition* **108**, 804-809 (2008).

5. S. Rossi *et al.*, Safety and recommendations for TMS use in healthy subjects and patient populations, with updates on training, ethical and regulatory issues: Expert Guidelines. *Clinical Neurophysiology* **132**, 269-306 (2021).

6. P. M. Rossini *et al.*, Non-invasive electrical and magnetic stimulation of the brain, spinal cord, roots and peripheral nerves: Basic principles and procedures for routine clinical and research application. An updated report from an I.F.C.N. Committee. *Clinical Neurophysiology* **126**, 1071-1107 (2015).

7. I. Berent *et al.*, Role of the motor system in language knowledge. *Proceedings of the National Academy of Sciences* **112**, 1983-1988 (2015).

**Appendix**

**The materials in Experiment 2**

|  | Monosyllables | | Disyllables | |
| --- | --- | --- | --- | --- |
| Item | **Stop-nasal** | **Sonorant-stop** | **Stop-nasal** | **Sonorant-stop** |
| 1 | cnim | lpim | cenim | lepim |
| 2 | cnek | rtek | cenek | retek |
| 3 | dlif | rdif | delif | redif |
| 4 | dlof | rdof | delof | redof |
| 5 | dmip | mdip | demip | medip |
| 6 | dmup | mdup | demup | medup |
| 7 | dnup | rdup | denup | redup |
| 8 | dnish | rbish | denish | rebish |
| 9 | gmep | lgep | gemep | legep |
| 10 | gmon | lfon | gemon | lefon |
| 11 | gmef | rgef | gemef | regef |
| 12 | gmit | mgit | gemit | megit |
| 13 | kmef | lkef | kemef | lekef |
| 14 | kmaf | rgaf | kemaf | regaf |
| 15 | cnik | rkik | cenik | rekik |
| 16 | cnuk | mcuk | cenuk | mecuk |
| 17 | cmup | ltop | cemup | letop |
| 18 | cmep | rkep | cemep | rekep |
| 19 | pnik | ltik | penik | letik |
| 20 | pnaf | rpaf | penaf | repaf |
| 21 | tluf | rtuf | teluf | retuf |
| 22 | tlep | mtep | telep | metep |
| 23 | tnok | rtok | tenok | retok |
| 24 | tmaf | mtaf | temaf | metaf |
| 25 | tnef | rtef | tenef | retef |
| 26 | tnuk | mguk | tenuk | meguk |
| 27 | tmap | rpap | temap | repap |
| 28 | tmok | mtok | temok | metok |
